# Influence of Polymorphism on the *NFkB1* Gene (rs28362491) on the Susceptibility to Sarcopenia in the Elderly of the Brazilian Amazon

**DOI:** 10.3390/jpm11101045

**Published:** 2021-10-19

**Authors:** Esdras E. B. Pereira, Darlen C. de Carvalho, Luciana P. C. Leitão, Juliana C. G. Rodrigues, Antônio A. C. Modesto, Evitom C. de Sousa, Sidney E. B. dos Santos, Marianne R. Fernandes, Ney P. C. dos Santos

**Affiliations:** 1 Laboratory of Human and Medical Genetics, Institute of Biological Science, Federal University of Pará, Belém 66077-830, Pará, Brazil; esdrasedgarbp@gmail.com (E.E.B.P.); antonioacm@ufpa.br (A.A.C.M.); sidneysantos@ufpa.br (S.E.B.d.S.); 2 Oncology Research Center, Federal University of Pará, Belém 66073-005, Pará, Brazil; darlen.c.cavalho@gmail.com (D.C.d.C.); colaresluciana@gmail.com (L.P.C.L.); julianacgrodrigues@gmail.com (J.C.G.R.); fernandesmr@yahoo.com.br (M.R.F.); 3 Resistance Exercise and Health Laboratory, State University of Pará, Belém 66095-049, Pará, Brazil; evitomuepa@gmail.com

**Keywords:** skeletal muscle, genetic biomarkers, *NFkB1*, sarcopenia

## Abstract

Background: Sarcopenia is a disease characterized by progressive reduction in muscle mass and strength or function. Although it is known that sarcopenia may be associated with environmental factors, studies suggest the identification of genes related to skeletal muscle maintenance that explain the susceptibility to the disease. Objective: To analyze the influence of *NFkB1* gene polymorphism on susceptibility to sarcopenia in the elderly. Methods: This is a case-control study, which included 219 elderly people, 74 elderly people with sarcopenia, and 145 without sarcopenia. Samples were analyzed for *NFkB1* gene polymorphism (rs28362491), genotyped in PCR, and followed by fragment analysis. To avoid misinterpretation due to population substructure, we applied a previously developed set of 61 informative ancestral markers that were genotyped by multiplex PCR. We used logistic regression to identify differences in genotypic frequencies between elderly people with and without sarcopenia. Results: It was observed that the *NFkB1* gene polymorphism presented frequencies of 24%, 50%, and 26% for the genotype DEL/DEL, DEL/INS, and INS/INS, respectively. Furthermore, elderly individuals with the INS/INS genotype had increased chances (*p* = 0.010; OR:2.943; 95%CI:1.301–6.654) for the development of sarcopenia. Conclusion: The INDEL polymorphism of the *NFkB1* gene (rs28362491) may influence the susceptibility to sarcopenia in the elderly in elderly people in the Amazon.

## 1. Introduction

Sarcopenia is a progressive degenerative disease characterized by loss of skeletal muscle mass and reduced function [1]. Worldwide, this disease is common between 9.9% and 40.4% of people over 60 years, depending on the population investigated and the criteria used [2]. In Brazil, this prevalence is 17% among the elderly [3]. The clinical consequences of sarcopenia include the risk of falls, fractures, loss of function, frailty, and increased mortality, generating significant personal and socioeconomic costs, and it is considered a public health problem that needs to be identified early [4,5,6].

The pathophysiology of sarcopenia is complex and can be attributed to a variety of factors, including oxidative stress, inflammation, apoptosis, and mitochondrial dysregulation [7]. The disease is also associated with environmental factors, such as physical activity and diet, and may have specific genetic influences, related to skeletal muscle maintenance, which may explain the variability of inter-individual characteristics related to muscle mass and strength [8].

This has motivated studies that suggest the importance of determining the genetic factors that are associated with aging and that can modulate skeletal muscle phenotypes in the elderly and the unfavorable genotypes associated with accelerated sarcopenia [9]. These would be biomarkers, possibly used in screening, diagnosis, staging, and sarcopenia prognosis [10,11,12].

Most studies involving genetic susceptibility to sarcopenia explore genes for hormones, receptors, cytokines, growth factors, structural factors, and metabolism [10,11,13]. Despite this, little progress has been made in identifying the specific genetic influences on sarcopenia. Furthermore, there are few studies that aim to predict the risk of sarcopenia based on single nucleotide polymorphisms (SNPs) [8]. Thus, further studies in different populations are needed to understand the genetic influence on sarcopenia. 

A common element of these pathways is nuclear factor kappa B (NFkB), a family of pleiotropic transcription factors, NFkB1 (p50/p105), NFkB2 (p52/p100), RelA (p65), c-Rel and Rel B, which regulate other myogenic transcription factors such as MyoD, MURF-1, and MAXbx involved in the development of sarcopenia [14,15,16].

The *NFkB1* gene is located on chromosome 4q24 and consists of 24 exons [16]. The *NFkB1* gene polymorphism (rs28362491) occurs between activator protein 1 (AP-1) and the κB binding site and has been associated with diseases of aging, such as inflammatory diseases, believed to also influence longevity [17,18,19,20]. Therefore, the aim of this study was to investigate possible associations between *NFkB1* gene polymorphism (rs28362491) and susceptibility to sarcopenia in the elderly people in the Amazon.

## 2. Methods

### 2.1. Ethical Conformity

The study was approved by the Research Ethics Committees of the Oncology Research Center, under protocol 927.808/2014, and by the João de Barros Barreto University Hospital, under protocol number 941.207/2015. All participants signed an informed consent form.

### 2.2. Case and Control

Participants were randomly selected from two public health care centers in the city of Belém do Pará, in the Brazilian Amazon, in a retrospective case-control study design. The 219 individuals were 60 years old or older, of both sexes, not belonging to the same family nucleus, and of the same socioeconomic level. Two groups were structured: 74 patients with sarcopenia (Case Group) and 145 patients without any type of sarcopenia (Control Group).

### 2.3. Sarcopenia Assessment

The sarcopenia diagnosis was based on the EWGSOP algorithm, following two criteria: mass reduction and physical performance [21]. The equation by Lee et al. [22] was used to estimate total muscle mass (MMT): MMT = (0.244 × body weight) + (7.8 × height) + (6.6 × gender) − (0.098 × age) + (race − 3.3). The number 0 was assigned for women and 1 for men, −1.2 for Asians, 1.4 for blacks, and 0 for whites. The race variable was based on the predominance of genomic ancestry. This equation was validated for the Brazilian population using DEXA as the “gold standard”. The equation was fitted by dividing by height squared, creating a total muscle mass index (TMMI). The cutoff point used to determine reduced muscle mass was ≤6.75 kg/m^2^ for women and ≤10.75 kg/m^2^ for men. Physical performance was assessed by mobility, using the 6-m Gait Speed Test (TVM6) and the cutoff point of <0.8 m/s indicating participants with low physical performance [1,21].

### 2.4. Clinical Assessment

The clinical assessment consisted of scales and quantitative tests. The clinical characterization of the sample included sex, age, lifestyle (alcoholism, smoking, and sedentary lifestyle), present comorbidities, nutritional status, and a screening questionnaire for sarcopenia (SARC-CalF) and anthropometry. The presence of comorbidities was assessed using the Charlson Comorbidity Index (ICC) [23]. Nutritional status was assessed by the Mini Nutritional Assessment (MAN) [24]. The SARC-CalF questionnaire consists of 6 items questioning strength, help to walk, stand up from a chair, climbing stairs, falls, and calf perimeter [25]. Anthropometry includes measurement of weight (kg), height (m), body mass index (kg/m^2^), and calf perimeter (cm).

### 2.5. DNA Extraction and Quantification

Extraction of genomic DNA from peripheral blood leukocytes was conducted using a Mini Spin Plus Kit (P.250, Biopur, Biometrix) according to the manufacturer’s recommendations. DNA concentration and purity were measured with a NanoDrop 1000 spectrophotometer (Thermo Scientific NanoDrop 1000; NanoDrop Technologies).

### 2.6. Genotyping

The *NFkB1* gene polymorphism (rs28362491) was genotyped by a multiplex PCR reaction followed by capillary electrophoresis. For the amplifications, 0.5 µL of the QIAGEN Multiplex PCR Kit (QIAGEN), 1.0 µL of Q-solution, 1.0 µL of Primer Mix, 2.0 µL of water, and 20 ng of DNA were used. PCR was performed following the following protocol: an initial denaturation at 95 °C for 15 min, 35 cycles at 94 °C for 45 s, 60 °C for 90 s, and 72 °C for 60 s, followed by a final extension of 70 °C for 30 min. Analysis of the PCR amplicons was performed from an electrophoresis using the ABI Prism 3130 sequencer and GeneMapper ID v.3.2 software.

### 2.7. Hardy–Weinberg Equilibrium Analysis (HWE)

The allelic and genotypic frequency of the polymorphism was determined by direct counting of alleles, and then Hardy–Weinberg equilibrium (HWE) was calculated using Arlequin 3.5.1.2 software. The individual proportions of European, African, and Amerindian genetic ancestry were estimated using Structure 2.3.3 software. It was evidenced that the polymorphism was present in the HWE.

### 2.8. Genetic Ancestrality Analysis

Genotyping was performed to analyze the ancestry of the samples, performed according to Ramos et al. [26], using 61 informative markers of autosomal ancestry in three multiplex PCR reactions. Amplicons were analyzed by electrophoresis using the ABI Prism 3130 sequencer and GeneMapper ID v.3.2 software. The individual proportions of European, African, and Amerindian genetic ancestry were estimated using Structure v.2.3.3 software, assuming three parental populations.

### 2.9. Statistical Analysis

For the comparative analysis between the studied groups, Pearson’s Chi-Square Test was applied, for categorical variables, and the Mann–Whitney test for non-parametric quantitative variables. To analyze the risk of polymorphisms on sarcopenia, a logistic regression controlled by the covariates age, comorbidity, sedentary lifestyle and nutritional status was performed. All statistical analyses were performed using the statistical package of the SPSS 20.0 software, respecting the significance level of 5% (*p*-value ≤ 0.05).

## 3. Results

A prevalence of sarcopenia of 34% of the elderly evaluated was observed, being more frequent in women (56.8%), aged over 70 years (56.8%), with a greater number of comorbidities, with a history of sedentary lifestyle (90.5%), and possessing a predominantly European ancestry. It was evident that the demographic and clinical characteristics between the groups differed significantly for age (*p* = 0.014), comorbidities (*p* < 0.0001), sedentary lifestyle (*p* < 0.0001), nutritional status (*p* < 0.0001), and the SARC-CalF score (*p* < 0.0001) (Table 1).

Table 2 shows information regarding anthropometry, total muscle mass index, and functional mobility. In anthropometry, it could be observed that the elderly group with sarcopenia was lighter in weight (*p* < 0.0001), with lower BMI (*p* < 0.0001), and lower calf circumference (*p* < 0.0001). In addition, this group also had a lower total muscle mass index (*p* < 0.0001) and lower gait speed (*p* < 0.0001).

It was evidenced that polymorphism was present in the HWE (*p*-value 0.955). The results of the logistic regression analysis showed that the INS/INS genotype of the *NFkB1* polymorphism (rs28362491) increased the risk of sarcopenia compared to the other genotypes (OR: 2.943; 95% CI: 1.301–6.654). As for allele frequency, the INS allele of this polymorphism increased the risk of sarcopenia compared to the DEL allele (OR: 1.932; 95%CI: 1.187–3.145) (Table 3).

Comparisons of total muscle mass index (TMMI), calf circumference, and gait speed among the three NFKB1 genotypes (rs28362491) shown in Table 4 show that the calf circumference was significantly smaller among the elderly with the INS/INS genotype (*p*-value: 0.013). The total muscle mass index and gait speed were also lower in the elderly with the INS/INS genotype; however, this difference was not significant (Table 4).

## 4. Discussion

Sarcopenia is a common syndrome among the elderly, and its prevalence varies significantly worldwide [27,28]. In the present study, in the Amazon region, the prevalence of sarcopenia was 34%, similar to a study carried out in the southern region of Brazil, which identified a prevalence of 33.3% among the elderly [29]. However, both are superior to what was observed by Diz et al. [3], who identified in their review an average prevalence of 17% of sarcopenia among elderly Brazilians. These distinctions must be analyzed with caution, due to the use of different methods for assessing sarcopenia between studies and differences in the characteristics of the populations investigated.

In this study, most elderly people with sarcopenia were over 70 years of age (56.8%) and were female (56.8%). According to Cruz-Jentoft et al. [21], the frequency of sarcopenia varies from 5 to 13% in the elderly aged between 60 and 70 years and increases to 11–50% for those with more advanced age, which may be associated with the identification of a higher prevalence of sarcopenia in this population.

As in the rest of the world, in Brazil the life expectancy of the female population is greater than that of men, causing them to experience more geriatric syndromes, which may justify sarcopenia being more prevalent in this group [3,30,31]. Furthermore, the emergence of sarcopenia in women seems to be associated with menopause, which causes hormonal changes, which modify the maintenance of muscle tissue [32]. This reduction in sex hormones is more accelerated among women than men [27].

Susceptibility to sarcopenia includes constitutional risk factors and lifestyle and health conditions. Among the constitutional factors are female gender and genetic propensity [33]. Genetic influence explains a significant fraction of the inter-individual variability of sarcopenia, which is driven by genes involved in pathways of metabolism, DNA damage repair, oxidative stress, inflammatory and immune responses, and programmed cell death [34].

In the present study, the analysis of the *NFkB1* gene polymorphism (rs28362491) showed relevant results. The polymorphism presented frequencies of 24%, 50%, and 26% for the genotype DEL/DEL, DEL/INS, and INS/INS, respectively. Elderly individuals with the INS/INS genotype had increased chances (*p* = 0.010; OR: 2.943; 95%CI: 1.301–6.654) for the development of sarcopenia. Therefore, individuals homozygous for this insertion in the *NFkB1* gene have an approximately three-fold increased risk for the development of sarcopenia. In addition, the elderly with this genotype had smaller calf circumference (*p* = 0.013), which also indicates reduced muscle mass. 

Oxidative stress, immune response and chronic inflammation influence the maintenance of skeletal muscle, affecting the balance between protein synthesis and breakdown, inducing muscle wasting [14]. The INS/INS genotype of the rs28362491 polymorphism of the *NFkB1* gene has been related to the risk of developing inflammatory diseases associated with altered immune response [16]. The likely mechanism that explains this association may be related to the increased expression and activity of NFkB1, where the INS allele is supposedly associated with increased promoter activity and increased expression of NFkB mRNA [34].

The increased activity of NFkB1 promotes the expression of proteins of the ubiquitin–proteasome system, involved in the breakdown of muscle proteins, in the increased expression of molecules related to inflammation, and in the influence of the myogenic differentiation process, which is necessary for the regeneration of skeletal muscles [35,36]. These pathways, when activated, favor chronic inflammation, sarcopenia, and the frailty syndrome [37,38,39].

This shows that sarcopenia is certainly related to genetic and environmental mechanisms, which manifest as atrophy, reduced endurance, and loss of muscle strength, which culminate in functional disabilities [40]. This is because joint movement needs preserved muscle strength to maintain gait, limb movements, and actions against gravity, such as sitting and standing, necessary for activities of daily living [41,42].

We believe that the results of association of the *NFKB1* gene polymorphism (rs28362491) with sarcopenia observed in the present study can also be found in other Brazilian regions, respecting the constitutional characteristics of populations between regions. According to Souza et al. [43] the proportions of European, African, and Amerindian genomic ancestry are 68.1%, 19.6%, and 11.6%, respectively, while in the states of the Amazon region, these respective proportions are 52.6%, 19.8%, and 27.7%, revealing that the Amazon region has a greater contribution from Amerindian genomic ancestry, similar to what was observed in the present study.

In the world, as it is a germline structural variant, similar results of this association of *NFKB1* gene polymorphism (rs28362491) with sarcopenia can be found, considering that this polymorphism has already been described in non-Brazilian populations [17,18,19,20]. There are already studies showing the association of this gene with the muscle [14,15,16]. However, our study is the first to show an association of this polymorphism with sarcopenia.

The NFkB1 pathway is not only activated in aging, but directly contributes to age-related pathologies such as sarcopenia. Knowledge of genetic polymorphisms, present in this and other pathways, is important in translational research for sarcopenia, as they provide information for the identification of susceptibility, risk stratification, and potential therapeutic targets, whether by pharmacological or non-pharmacological measures, which aim to maintain or increase the mass, strength, and function of the skeletal muscle, thus preserving the functional capacities and independence of the elderly.

## 5. Conclusions

In conclusion, the *NFkB1* gene variant (rs28362491) was associated with the risk of developing sarcopenia in elderly people in the Amazon. Elderly people with the INS/INS genotype were almost three times more susceptible to developing the disease. This indicates its potential additional use in screening for sarcopenia in the elderly population, which may facilitate the targeting of prevention, control, and treatment strategies for the disease.

## Figures and Tables

**Table 1:** Demographic and clinical characteristics of the investigated groups.

Characteristics	Case (*n* = 74)	Control (*n* = 145)	*p*-Value
**Sex**			
Male	32 (43.2%)	51 (35.2%)	0.2440 ^a^
Female	42 (56.8%)	94 (64.8%)	
**Age**			
≤70 years	32 (43.2%)	88 (60.7%)	0.0140 ^a,^*
>70 years	42 (56.8%)	57 (39.3%)	
**Charlson Index (Score)**			
Median (p25%–p75%)	5.0 (4.0–7.0)	3.0 (2.0–5.0)	<0.0001 ^b,^*
**Lifestyle**			
Smoking	46 (62.2%)	74 (51.0%)	0.1180 ^a^
Alcoholism	34 (45.9%)	74 (51.0%)	0.4760 ^a^
Sedentary lifestyle	67 (90.5%)	50 (34.5%)	<0.0001 ^a,^*
**Nutritional Status (NMA)**			
Normal	49 (66.2%)	139 (95.9%)	<0.0001 ^a,^*
Malnourished	25 (33.8%)	6 (4.1%)	
**SARC-CalF (Score)**			
Median (p25%–p75%)	16.0 (12.0–18.0)	10.0 (0.0–10.0)	<0.0001 ^b,^*
**Ancestry**			
European	0.451 ± 0.178	0.456 ± 0.170	0.9530 ^b^
Amerindian	0.316 ± 0.151	0.299 ± 0.149	0.4200 ^b^
African	0.232 ± 0.123	0.244 ± 0.146	0.8470 ^b^

NMA, Nutritional Mini-Assessment; SARC-CalF, Screening Questionnaire for Sarcopenia; p, percentile. ^a^ Pearson’s Chi-Square test; ^b^ Mann–Whitney test; * *p*-value ≤ 0.05.

**Table 2:** Anthropometry, total muscle mass index, and functional mobility of the investigated groups.

Variables	Case (*n* = 74)	Control (*n* = 145)	*p*-Value ^a^
**Anthropometry**			
Weight (kg)	50.99 ± 11.31	65.99 ± 11.89	<0.0001 *
Height (m)	1.58 ± 0.09	1.59 ± 0.09	0.8890
BMI (kg/m^2^)	20.31 ± 4.52	26.08 ± 4.57	<0.0001 *
Calf Circumference (cm)	27.92 ± 2.09	34.26 ± 3.00	<0.0001 *
**Total Muscle Mass Index (kg/m^2^)**			
Mean (±sd)	7.17 ± 1.69	8.50 ± 1.59	<0.0001 *
**Functional Mobility**			
Gait Speed (m/s)	0.30 ± 0.19	0.60 ± 0.32	<0.0001 *

BMI, body mass index; sd, standard deviation. ^a^ Mann–Whitney test; * *p*-value ≤ 0.05.

**Table 3:** Genotypic distribution of the *NFkB1* gene polymorphism (rs28362491) in the investigated groups.

Genotype	Case (*n* = 74)	Control (*n* = 145)	*p*-Value ^a^	OR (95% CI)
***NFkB1* (rs28362491)**				
DEL/DEL	15 (20.3%)	37 (25.5%)	0.010 *	INS/INS vs. Others: 2.943 (1.301–6.654)
DEL/INS	30 (40.5%)	79 (54.5%)		
INS/INS	29 (39.2%)	29 (20.0%)		
Allele DEL	60 (40.5%)	153 (52.8%)	0.008 *	1.932 (1.187–3.145)
Allele INS	88 (59.5%)	137 (47.2%)		

INS/INS, insertion/insertion; DEL/INS, deletion/insertion; DEL/DEL, deletion/deletion; OR, odds ratio; CI, confidence interval; ^a^ logistic regression adjusted for age, comorbidities, sedentary lifestyle, and nutritional status; * *p*-value ≤ 0.05.

**Table 4:** Comparison of total muscle mass index, calf circumference, and gait speed among the NFkB1 genotypes (rs28362491) of the investigated groups.

Variables	*NFkB1* Genotypes (rs28362491)				INS/INS vs. Others *p*-Value ^a^
	DEL/DEL (*n* = 52)	DEL/INS (*n* = 109)	INS/INS (*n* = 58)	DEL/DEL+DEL/INS (*n* = 161)	
**TMMI (kg/m^2^)**					
Mean (±sd)	8.39 ± 1.58	8.00 ± 1.77	7.84 ± 1.82	8.14 ± 1.71	0.227
**Calf Circumference (cm)**					
Mean (±sd)	31.98 ± 3.09	32.71 ± 4.23	31.05 ± 2.29	32.48 ± 3.91	0.013 *
**Gait Speed (m/s)**					
Mean (±sd)	0.43 ± 0.27	0.55 ± 0.34	0.46 ± 0.30	0.51 ± 0.33	0.269

TMMI, total muscle mass index; INS/INS, insertion/insertion; DEL/INS, deletion/insertion; DEL/DEL, deletion/deletion; sd, standard deviation; ^a^ Mann–Whitney test; * *p*-value ≤ 0.05.
